# Persistent low carriage of serogroup A *Neisseria meningitidis*two years after mass vaccination with the meningococcal conjugate vaccine, MenAfriVac

**DOI:** 10.1186/s12879-014-0663-4

**Published:** 2014-12-04

**Authors:** Paul A Kristiansen, Absatou Ky Ba, Abdoul-Salam Ouédraogo, Idrissa Sanou, Rasmata Ouédraogo, Lassana Sangaré, Fabien Diomandé, Denis Kandolo, Inger Marie Saga, Lara Misegades, Thomas A Clark, Marie-Pierre Préziosi, Dominique A Caugant

**Affiliations:** WHO Collaborating Center for Reference and Research on Meningococci, Norwegian Institute of Public Health, Oslo, Norway; Laboratoire National de Santé Public, Ouagadougou, Burkina Faso; Centre Hospitalier Universitaire Souro Sanou, Bobo-Dioulasso, Burkina Faso; Centre Hospitalier Universitaire Yalgado, Ouagadougou, Burkina Faso; Centre Hospitalier Universitaire Pédiatrique Charles de Gaulle, Ouagadougou, Burkina Faso; WHO Inter Country Support Team, Ouagadougou, Burkina Faso; Centers for Disease Control and Prevention, Atlanta, USA; Meningitis Vaccine Project, Ferney, France; WHO Initiative for Vaccine Research, Geneva, Switzerland; Faculty of Medicine, University of Oslo, Oslo, Norway

**Keywords:** Neisseria meningitidis, Carriage, Meningitis, Burkina Faso, Conjugate vaccine, MLST, Meningitis belt, MenAfriVac, Herd immunity

## Abstract

**Background:**

The conjugate vaccine against serogroup A *Neisseria meningitidis* (NmA), MenAfriVac, is currently being introduced throughout the African meningitis belt. In repeated multicentre cross-sectional studies in Burkina Faso we demonstrated a significant effect of vaccination on NmA carriage for one year following mass vaccination in 2010. A new multicentre carriage study was performed in October-November 2012, two years after MenAfriVac mass vaccination.

**Methods:**

Oropharyngeal samples were collected and analysed for presence of *N. meningitidis* (Nm) from a representative selection of 1-29-year-olds in three districts in Burkina Faso using the same procedures as in previous years. Characterization of Nm isolates included serogrouping, multilocus sequence typing, and *porA* and *fetA* sequencing. A small sample of invasive isolates collected during the epidemic season of 2012 through the national surveillance system were also analysed.

**Results:**

From a total of 4964 oropharyngeal samples, overall meningococcal carriage prevalence was 7.86%. NmA prevalence was 0.02% (1 carrier), significantly lower (OR, 0.05, P = 0.005, 95% CI, 0.006-0.403) than pre-vaccination prevalence (0.39%). The single NmA isolate was sequence type (ST)-7, P1.20,9;F3-1, a clone last identified in Burkina Faso in 2003. Nm serogroup W (NmW) dominated with a carriage prevalence of 6.85%, representing 87.2% of the isolates. Of 161 NmW isolates characterized by molecular techniques, 94% belonged to the ST-11 clonal complex and 6% to the ST-175 complex. Nm serogroup X (NmX) was carried by 0.60% of the participants and ST-181 accounted for 97% of the NmX isolates. Carriage prevalence of serogroup Y and non-groupable Nm was 0.20% and 0.18%, respectively. Among the 20 isolates recovered from meningitis cases, NmW dominated (70%), followed by NmX (25%). ST-2859, the only ST with a serogroup A capsule found in Burkina Faso since 2004, was not found with another capsule, neither among carriage nor invasive isolates.

**Conclusions:**

The significant reduction of NmA carriage still persisted two years following MenAfriVac vaccination, and no cases of NmA meningitis were recorded. High carriage prevalence of NmW ST-11 was consistent with the many cases of NmW meningitis in the epidemic season of 2012 and the high proportion of NmW ST-11 among the characterized invasive isolates.

**Electronic supplementary material:**

The online version of this article (doi:10.1186/s12879-014-0663-4) contains supplementary material, which is available to authorized users.

## Background

Bacterial meningitis in sub-Saharan Africa has mainly been caused by *N. meningitidis* (Nm) with localized outbreaks or large epidemics occurring every year in the dry season [1]–[4]. The number of cases usually increases in January and drops in April-May with the first rainfall.

Nm is normally colonising the upper respiratory tract in humans without causing disease [3],[5],[6]. The bacteria circulate in the human population by transmitting from person to person through droplets and close contact. However, occasionally Nm may infect the blood and cause meningitis and/or septicaemia, with a possible fatal outcome occurring within hours.

Nm is classified into serogroups according to the structure of its polysaccharide capsule. In the meningitis belt, epidemics have essentially been caused by Nm of serogroups A (NmA), but serogroups W (NmW) and X (NmX) have also caused large outbreaks [7]–[9].

As the capsule is immunogenic and probably the most important virulence factor, capsular polysaccharide of serogroups A, C, W and Y has been used as vaccine antigens. Until very recently, A/C and A/C/W polysaccharide vaccines have been the only vaccines available for use in sub-Saharan Africa. Due to the relative short protection provided by polysaccharide vaccines and their inability to elicit a good antibody response in children below 2 years of age, these vaccines have been used mainly to stop ongoing outbreaks [10]. Following the success of *Haemophilus influenzae* type b and pneumococcal conjugate vaccines, Nm vaccines coupling a carrier protein to the main disease-causing polysaccharide serogroups were developed. In addition to eliciting strong and long lasting immune response in children below 2 years [5],[11], conjugate vaccines have also been shown to confer herd protection by reducing the carriage prevalence, thus interrupting transmission [12]–[16].

A monovalent serogroup A tetanus toxoid-conjugated vaccine, MenAfriVac, has been developed with the goal of eliminating the devastating NmA epidemics in Africa [17]–[19]. The vaccine was shown to be safe and immunogenic [20] and its low price made it appropriate for routine vaccination and mass vaccination campaigns in Africa. MenAfriVac was prequalified by the World Health Organization (WHO) in June 2010 to be given as a single dose in the age group 1–29 years.

Being a conjugate vaccine, it was hoped that MenAfriVac will have an effect on NmA carriage, similarly to what has been found in the UK following vaccination campaign with a monovalent serogroup C conjugate on NmC carriage [14],[15]. Given the fact that not all age-groups were eligible to receive the vaccine, impact of vaccination on meningococcal carriage would be beneficial for the whole population by reducing transmission. To demonstrate the impact of this new vaccine in an African setting, meningococcal carriage studies were planned ahead of MenAfriVac implementation in the meningitis belt.

Burkina Faso was the first country to vaccinate the whole 1-29-year-old population in 2010 [21]. Mali and Niger introduced the vaccine in 2010–2011 and other countries of the meningitis belt rapidly followed; over 150 million people had received the vaccine by the end of 2013. The impact of MenAfriVac on NmA disease and carriage, and the evidence of herd immunity, were first demonstrated in Burkina Faso. The risk for meningitis significantly decreased in all age groups, not only in the 1–29 year-old vaccinated population, and based on laboratory-confirmed cases, the risk for NmA meningitis was reduced by 99.8% [22]. Up to 13 months after vaccination, NmA carriage remained undetected among both vaccinated and unvaccinated populations [23] and there was no evidence of capsule replacement of the ST-2859 clone previously responsible for NmA disease and carriage in Burkina Faso [24]. Similar results were recently reported from Chad that experienced an epidemic in 2010; outbreaks of NmA meningitis occurred only in non-vaccinated districts and NmA carriage was significantly lower 4–6 months after vaccination [25]. As the vaccine is not yet introduced in childhood vaccination programs, the impact of this public health intervention relies in large part on the herd protection afforded by the vaccine. It is thus important to document how long this protection will last through continued laboratory-based surveillance and follow-up studies on carriage.

To study the long term impact of MenAfriVac vaccination on carriage, an existing multicentre carriage study in Burkina Faso was extended and conducted in October-November 2012 using the same protocol as in previous years. Results from this carriage sampling together with molecular characterization of invasive isolates collected through surveillance are presented here.

## Methods

### Ethics

The carriage study obtained ethical clearance from the Norwegian Regional Committee for Medical Research Ethics, Southern Norway, the Ethical Committee for Health Research in Burkina Faso and the Institutional Review Board at the Centers for Disease Control and Prevention (CDC), Atlanta, USA. Informed written consent was obtained from the participants or from their parent or guardian if the participant was < 18 years. The invasive isolates were collected within the national surveillance system and did not require ethical approval.

### Carriage study design and oversight

The study was a continuation of a previously described multicentre repeated cross-sectional survey in three health districts in Burkina Faso [19]. Data presented here were collected during the tenth cross-sectional survey, designated Sampling 10 (S10). During four weeks, between the 15th of October and 11th of November 2012, we conducted a study of oropharyngeal carriage among 1-29-year-olds, in the urban district of Bogodogo and the two rural districts of Dandé and Kaya. MenAfriVac was first introduced in Kaya in mid-September 2010, while the rest of the country, including Bogodogo and Dandé introduced the vaccine in early December 2010. We consider this sampling campaign to be two years after vaccination for all three districts (range 23–25 months).

Using a multistage cluster design we randomly selected households in each district in which all healthy 1-29-year-olds were invited to participate, whether or not they had previously been vaccinated with MenAfriVac. Informed consent was obtained from each participant or guardian if the subject was < 18 years. Demographic data and MenAfriVac vaccination status for each participant was collected on personal digital assistants (PDAs).

### Collection of carriage isolates

The procedures for sampling and laboratory analysis have been described elsewhere [26],[27]. Oropharyngeal samples were collected in the field by swabbing the posterior pharyngeal wall and one tonsil. The samples from Bogodogo were analyzed at the Centre Hospitalier Universitaire Pédiatrique Charles de Gaulle in Ouagadougou, the samples from Dandé at the Centre Hospitalier Universitaire Souro Sanou in Bobo-Dioulasso and the samples from Kaya at the Centre Hospitalier Régional in Kaya.

The laboratory analyses included examination of colony morphology, oxidase test, Gram staining, enzymatic tests for detection of β-galactosidase (ONPG) and γ-glutamyltransferase (GGT) and serogrouping by slide agglutination, as previously described [26]. Oxidase-positive, Gram-negative diplococcic, ONPG-negative and GGT-positive isolates were considered as presumptive Nm and shipped to the Norwegian Institute of Public Health (NIPH), Oslo, Norway, for confirmatory analyses and molecular characterization.

### Laboratory quality control

A laboratory quality control (QC) system for assessing the quality of the carriage study in Burkina Faso was followed [27]. It included control of reagents, media and incubation conditions (internal QC), and the re-analysis of a subset of Nm-negative samples taken from two critical analytical steps: the identification of Nm colonies growing on the selective agar plate based on colony morphology, and the enzymatic tests (external QC). External QC was performed by NIPH and enabled the assessment of sensitivity and specificity of laboratory analysis using standard formulas, as described [27].

### Data management and statistical analyses

Demographic data from participants in the carriage study and laboratory data from Burkina Faso and Norway were merged into a combined database in Access (Microsoft Corporation, Redmond, WA). Samples without complete traceability, due to missing or duplicated links between the laboratory result and the demographic data of the participant, were excluded from the analysis.

During recruitment of participants we asked whether or not they had been vaccinated with MenAfriVac. For the estimation of vaccine coverage, those responding “no” or “don’t know” were considered as not vaccinated in order to present the most conservative coverage estimate.

Data analysis was performed in STATA 12 [28] and Excel 2010 (Microsoft Corporation, Redmond, WA). Bivariate analysis was done using odds ratio (OR) and 95% confidence interval (CI) calculations corrected for the cluster sampling design.

### Collection of invasive isolates

Among 843 culture-positive cerebrospinal fluid (CSF) isolates collected during the 2012 epidemic season, 20 that had been processed at the Centre Hospitalier Universitaire Pédiatrique Charles de Gaulle were sent in Trans-isolate medium [29] to the WHO Collaborating Centre for Reference and Research on Meningococci at NIPH. Fifteen were culture-positive on arrival at NIPH.

### Strain characterization

DNA from each Nm strain was extracted by suspending 1 loop of bacteria in 200 μl Tris-EDTA (TE) buffer, pH 8.0, heating at 95°C for 10 minutes, centrifugation at 16,000 × g for 5 min and storing the supernatant at −20°C until use.

For the carriage isolates and culture-positive invasive isolates, molecular characterization consisted of the determination of the sequence type (ST) by multilocus sequence typing (MLST) [30], and the variant of outer membrane protein PorA and FetA by DNA sequencing of the *porA* and *fetA* genes [31],[32], using the primers recommended on the MLST website (http://pubmlst.org/neisseria). For isolates found to be non-serogroupable (NG) by slide agglutination, the serogroup was determined by capsule-gene PCR [33].

Invasive isolates were further characterized by antibiotic susceptibility to ciprofloxacin, ceftriaxone, rifampicin, penicillin G, sulphonamides, tetracycline and chloramphenicol. Each isolate was classified according to the European Committee on Antibiotic Susceptibility Testing breakpoints (www.eucast.org) based on minimal inhibitory concentration (MIC) to each antibiotic as determined by Etest (Biomérieux, France).

For culture-negative invasive isolates, the characterization was restricted to capsule-gene PCR and the determination of PorA variant by DNA sequencing of the *porA* gene using a nested *porA*-PCR [34].

## Results

### The carriage study

#### Study population and samples

The carriage study resulted in the collection and analysis of 4982 samples. Of these, 18 samples were excluded due to missing or duplicated links to the demographic data of the person swabbed. Thus, the total sample sizes used for the analysis were 4964, 1632 from Bogodogo, 1655 from Dandé and 1677 from Kaya. None of the excluded samples were Nm-positive.

The proportion of males was 43.4%, and 53.6% of all the participants were between 1 and 9 years of age. Based on the questionnaire, MenAfriVac vaccination coverage was 78.8% across all age groups, with 54.5% confirmed by presentation of the vaccination card. Vaccination coverage varied by age. Although none of the children below 2 years of age were old enough to be vaccinated in December 2010, 6.3% and 16.2% of the 1 and 2 years old, respectively, were reported to have received the vaccine. Coverage in the 3-year-olds was 61.1% while coverage in the 4-29-year-olds ranged from 78.5-96.6%.

### Meningococcal carriage by serogroup and ST

Of the 4964 participants included in the analysis, 390 (7.86%) were carriers of Nm. Carriage prevalence varied by district: prevalence was highest in Dandé (17.70%), followed by Kaya (3.34%) and Bogodogo (2.51%) (Table 1).

**Table 1:** **Meningococcal carriage in Burkina Faso two years after mass vaccination with MenAfriVac**

	District			
	Bogodogo (N = 1632)	Dandé (N = 1655)	Kaya (N = 1677)	Total (N = 4964)
Serogroup				
A	1 (0.06%)	0	0	1 (0.02%)
W	27 (1.65%)	284^a^ (17.16%)	29 (1.73%)	340 (6.85%)
X	5 (0.31%)	5 (0.30%)	20 (1.19%)	30 (0.60%)
Y	2 (0.12%)	2 (0.12%)	6 (0.36%)	10 (0.20%)
NG^b^	6 (0.37%)	2 (0.12%)	1 (0.06%)	9 (0.18%)
Total	41 (2.51%)	293 (17.70%)	56 (3.34%)	390 (7.86%)

^a^Of these, 105 isolates were randomly selected for molecular characterization.

^b^NG, Nongroupable *Neisseria meningitidis.*

One single NmA carrier was identified among the 4964 participants (0.02%). Compared to a pre-vaccination carriage prevalence of 0.39% [26], the measured prevalence was significantly lower (OR, 0.05, P = 0.005, 95% CI, 0.006-0.403). This strain was assigned to the ST-7 of the ST-5 clonal complex, with PorA;FetA variants P1.20,9;F3-1. The isolate came from a 13 year-old vaccinated boy living in the Bogodogo district. His vaccination status was confirmed by vaccination card. The family had received visitors from Ivory Coast shortly before the sample was taken. Within the boy’s household, three other persons aged 9, 18 and 21 years, were also included in the study: none of them were carriers.

NmW dominated in all three districts and accounted for 97%, 66% and 52% of the Nm isolates in the districts of Dandé, Bogodogo and Kaya, respectively. All the NmW carriage isolates from Bogodogo and Kaya were subjected to molecular characterization while of the 284 NmW isolates from Dandé, a subset of 105 isolates was characterized using molecular methods. The subset included every fourth isolate determined as NmW by slide agglutination and all NG by slide agglutination that were serogroup W by PCR. The majority of the characterized NmW isolates from all three districts were assigned to ST-11 of the ST-11 clonal complex. In Dandé as many as 98.1% of the NmW isolates were ST-11, while one isolate was ST-10171, a single locus variant of ST-11, and one isolate was ST-2881 of the ST-175 complex (Table 2). By extrapolating the characteristics of the subset of isolates from Dandé, we estimated that carriage prevalence of NmW ST-11 in Dandé was 16.7%. Prevalence of NmW ST-11 in Bogodogo and Kaya was 1.2% and 1.1%, respectively.

**Table 2:** **Molecular characteristics of meningococcal carriage isolates in the districts of Bogodogo (B), Dandé (D) and Kaya (K) in Burkina Faso two years after MenAfriVac vaccination**

Serogroup	ST-complex	ST no	PorA	FetA	No. of isolates	By district ^a^		
						B	D	K
A	5	7	P1.20,9	F3-1	1	1	0	0
W	11	11	P1.5,2	F1-1	126	19	86	21
	11	11	P1.5,2	F1-31	1	0	0	1
	11	11	P1.5,2	F3-56	1	0	0	1
	11	11	P1.5,2	F6-3	20	1	17	2
	11	9927	P1.5,2	F1-1	1	1	0	0
	11	10170	P1.5,2	F1-1	1	1	0	0
	11	10171	P1.5,2	F1-1	1	0	1	0
	175	2881	P1.5-1,2-36	F5-1	7	3	1	3
	175	9357	P1.5-1,2-36	F5-1	3	2	0	1
X	181	181	P1.5-1,10-1	F1-31	25	4	5	16
	181	181	P1.5-1,10-1	F1-147	1	0	0	1
	181	181	P1.5-1,10-1	F5-69	3	0	0	3
	181	10169	P1.5-1,10-1	F1-31	1	1	0	0
Y	167	767	P1.5-1,10-8	F1-3	1	0	0	1
	175	2881	P1. 21–14,28-3	F5-1	3	0	0	3
	23	1622	P1.5-1,2-2	F5-8	1	0	1	0
	23	4375	P1.5-1,2-2	F5-8	2	0	1	1
	UA^c^	192	P1.18-11,42-1	Neg.^d^	3	2	0	1
NG^b^	175	2881	P1.5-1,2-78	F5-1	2	0	2	0
	175	9357	P1.5-1,2-77	F5-1	1	1	0	0
	198	198	P1.18-24,25	F5-5	1	1	0	0
	UA^c^	192	P1.18-11,42-1	Neg.^d^	2	2	0	0
	UA^c^	4899	P1.21-14,28-3	F5-66	2	1	0	1
	UA^c^	8248	P1.21-14,28-3	F5-66	1	1	0	0

^a^Districts of Bogodogo (B), Dandé (D) and Kaya (K).

^b^NG, Nongroupable *Neisseria meningitidis.*

^c^UA, Unassigned to any clonal complex.

^d^Negative.

ST, Sequence Type; CC, Clonal complex.

NmX was the second most prevalent serogroup, found in 0.6% of the participants (Table 1). In Dandé this serogroup represented only 1.7% of the carriage isolates while in Bogodogo and Kaya the proportion was at 12.2% and 35.7%, respectively. All the 30 NmX isolates were assigned to the ST-181 complex, 29 (96.7%) to ST-181 and 1 (0.3%) to ST-10169 (Table 2).

Overall carriage of NmY and NmNG was low at 0.20% and 0.18%, respectively (Table 1). Serogroup Y was highest in Kaya (0.36%), while NmNG was highest in Bogodogo (0.37%).

**Figure 1:**
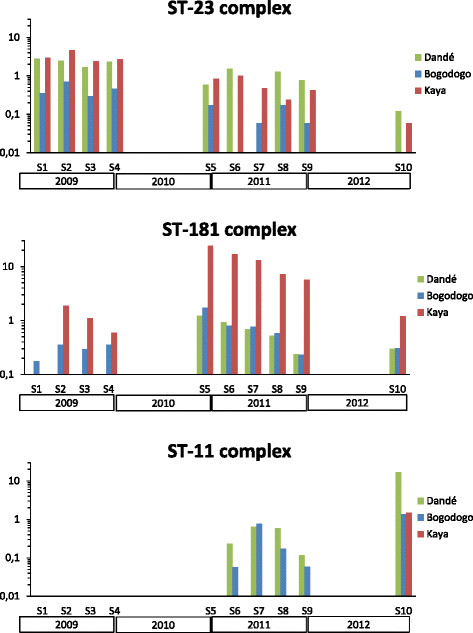
**Evolution of carriage prevalence for the dominant clonal complexes in Burkina Faso, 2009–2012.** The figure shows the carriage prevalence (%) of ST-23, ST-181 and ST-11 clonal complexes on a logarithmic scale in each study sites in Burkina Faso at ten sampling timepoints, S1-S10.

Comparisons of carriage prevalence of meningococci assigned to the ST-23, ST-181 and ST-11 clonal complexes with data from the three previous years are presented in Figure 1. Overall carriage of ST-11 associated isolates increased from 0.22% in 2011 to 6.61% in 2012 and the increase was highest in Dandé; from 0.39% to 16.98%. In contrast, carriage prevalence of meningococci assigned to ST-23 and ST-181 decreased from 2011 to 2012 (Figure 1).

### Meningococcal carriage by age and sex

**Figure 2:**
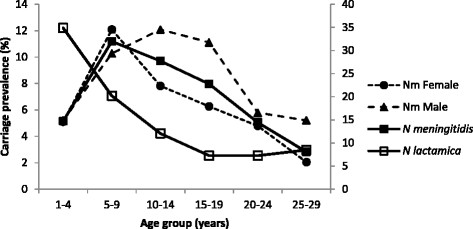
**Carriage prevalence of** ***N. meningitidis*** **by age and gender and prevalence of** ***N. lactamica*** **by age in Burkina Faso in 2012.** The figure shows carriage prevalence (%) of *N. meningitidis* by age and gender (left secondary axis) together with carriage prevalence (%) of *N. lactamica* by age (right secondary axis).

**Figure 3:**
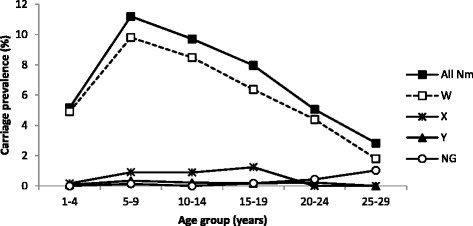
**Meningococcal carriage prevalence in Burkina Faso in 2012, by age and serogroup.** The figure shows meningococcal carriage prevalence (%) by age for all meningococci (Nm) and for serogroups W, X, Y and nongroupable (NG) meningococci.

Carriage prevalence was higher among male participants (8.72%) than females (7.19%) but the difference was not statistically significant when correcting for the cluster sampling design (P = 0.104). Prevalence varied with age with a peak at 11.20% in the 5-9-year-olds (Figure 2). We observed different age-related trends in carriage prevalence for males and females (Figure 2). For female participants, carriage was highest in the age group 5–9 years (12.09%) while for males the maximum carriage prevalence was found in 10-14-year-olds (12.09%) (Figure 2). The dominant NmW was the only serogroup showing a marked difference between age groups in terms of prevalence, because the other serogroups were represented by relatively few isolates (Figure 3).

### Carriage of *N. lactamica*

Isolates with Gram-negative diplococcic morphology, oxidase-positive and ONPG-positive reactions, belonging to the closely related species *N. lactamica*, were identified in 18.71% of the samples. Carriage was age-dependent with highest prevalence in 1–3 years old children (average, 36.5%, range 35.0-37.8%) and declining from the age of 3 years until the age of 19 years (Figure 2). Among participants ≥ 20 years, carriage of *N. lactamica* remained within a range of 7.2-8.5%, carriage among women was higher (range, 7.9%-9.0%) compared to males (range 1.1%-5.1%).

### Laboratory QC

QC samples (n = 270) that were determined as not harbouring Nm in Burkina Faso were re-analysed at NIPH; 150 were from the analytical step where colonies are picked from the primary agar plate for further analysis, based on colony morphology and 120 from the enzymatic ONPG and GGT tests. In total, 3 samples were identified as Nm and included in the data analysis: two isolates were from Bogodogo (NmW ST-11 and NmW ST-2881) and one isolate was from Dandé (NmW ST-11). When extrapolating the proportion of missed Nm in the two steps included in the QC to the total number of samples analysed, we estimated that 30 Nm may have been missed. Thus, the overall carriage rate of 7.86% was probably a slight underestimation and the prevalence should likely be corrected to 8.40%. Of the 404 presumptive Nm-positive samples sent to NIPH for confirmation and further characterisation, 387 were true positive (95.8%) while 17 were not Nm. Based on these numbers we calculated the sensitivity and specificity of meningococcal isolation and identification in Burkina Faso to be 92.8% and 99.6%, respectively.

### Clinical isolates

#### Sample collection

Of the 20 isolates sent to NIPH on trans-isolate medium, 15 were culture-positive upon arrival and characterized by serogrouping, MLST, *porA*/*fetA* sequencing (Table 3) and antibiotic susceptibility. The remaining 5 samples did not grow and were analysed by capsule gene PCR and *porA* sequencing.

**Table 3:** **Molecular characteristics of 20 meningococcal isolates from cerebrospinal fluid samples collected in Burkina Faso during the epidemic season of 2012**

Serogroup	Culture	ST-complex	ST no	PorA	FetA	No. of isolates
W	Pos.	11	11	P1.5,2	F1-1	9
	Pos.	11	2724	P1.5,2	F1-1	1
	Pos.	11	9766	P1.5,2	F1-1	1
	Neg.	ND	ND	P1.5,2	ND	3
X	Pos.	181	181	P1.5-1,10-1	F1-31	3
	Neg.	ND	ND	P1.5-1,10-1	ND	1
	Neg.	ND	ND	P1.7,16-46	ND	1
Y	Pos.	23	4375	P1.5-1,2-2	F5-8	1

ND, Not determined.

### Characteristics of invasive isolates

The 20 CSF isolates included 14 NmW, 5 NmX and 1 NmY. The NmW isolates all had PorA variant P1.5,2 and the culture-positive samples all belonged to the ST-11 clonal complex and were assigned to either ST-11, ST-2724 or ST-9766 (Table 3). The NmX isolates had either PorA variant P1.5-1,10-1 (4 of 5 isolates) or P1.7,16-46 (1 isolate). The 3 NmX isolates with a MLST result were assigned to ST-181 of the ST-181 clonal complex. The NmY isolate was assigned to ST-4375 of the ST-23 clonal complex. None of the clinical isolates were serogroup A or assigned to the ST-5 clonal complex, the only clonal complex responsible for NmA disease in sub-Saharan Africa in the past decades [8],[24],[35].

The 15 culture-positive isolates were susceptible to ciprofloxacin (MIC range, <0.002 – 0.008), ceftriaxone (MIC, < 0.002), rifampicin (MIC range, 0.008 – 0.25), penicillin G (MIC range, 0.032 – 0.125), tetracycline (MIC range, 0.125 – 0.25) and chloramphenicol (MIC range, 0.5 – 1.0). All the NmW strains were resistant to sulfamethoxazole (MIC > 1024) while the serogroups X and Y isolates were susceptible (MIC range, 0.5 – 2.0).

## Discussion

This study describes meningococcal carriage in Burkina Faso in October-November 2012, two years after the introduction of MenAfriVac. Molecular characteristics of the carriage isolates were compared to those of invasive isolates collected during the 2012 epidemic season. The significant reduction of NmA carriage after MenAfriVac vaccination still persisted. Serogroup W dominated both among carriage and patient isolates.

Although the small collection of invasive isolates that we analyzed was not a random selection, it reflected to some extent the characteristics of meningococcal disease isolates in Burkina Faso in 2012. Our collection was composed of 75% NmW and 20% NmX, which is comparable to the WHO surveillance data from the 2012 epidemic season showing that among the laboratory confirmed cases caused by Nm, 83.5% were NmW and 16.4% were NmX [36]. Analysis of a larger sample of invasive isolates collected from Burkina Faso in 2012 newly confirmed that all the NmW isolates belonged to the ST-11 complex [37]. A single NmY case was identified and this isolate was included in our strain collection. The surveillance system in Burkina Faso was strong in this period: of 6957 cases of suspect meningitis cases reported in 2012, 3292 CSF samples were collected, of which 1105 were reported with a laboratory result [36]. No cases of NmA meningitis were reported during the 2012, 2013 and 2014 epidemic seasons, before and after the carriage study [36].

This carriage study was the tenth sampling campaign performed in the same districts of Burkina Faso since 2009 [23],[24],[26],[27]. Efforts were made to keep all conditions as similar as possible to those in previous samplings. We used the same protocol, reagents, equipment and QC system. The teams were composed of essentially the same persons and retraining was conducted prior to start. The sensitivity and specificity of laboratory analysis in Burkina Faso, as extracted from the QC system, were comparable to previous campaigns [23],[26],[27] and show that variation of carriage prevalence was not due to variation in the quality of the analysis. Furthermore, the carriage prevalence of *N. lactamica* was within the range obtained from all the previous campaigns [38], providing yet another indication that the sampling was well conducted.

The age group included in the carriage study was the same as the age group targeted for vaccination in 2010. Since the entire country was vaccinated within a short timeframe, the carriage study was not designed with a non-vaccinated control group. However, since MenAfriVac was not part of the Expanded Program of Immunization and the study was performed two years after the national mass vaccination campaign, 21.2% of the study participants were considered non-vaccinated (17.6% reported to not be vaccinated; 3.6% did not remember), and 70% of the 1-3-year-olds were not vaccinated. The low vaccination coverage compared to previous studies [23],[39] and the age distribution of non-vaccinated was consistent with the time delay between mass-vaccination in 2010 and the survey. Immigration might also contribute to explain the low coverage but we did not collect such data. The reliability of the participants’ responses to whether they had been vaccinated with MenAfriVac is questionable, however, because the survey was conducted two years after vaccination and only 54.5% of those responding “yes” presented a vaccination card. A relatively small number of 1- and 2-year-olds were reported to have received MenAfriVac, despite the fact that they were not eligible for vaccination in 2010. It is possible that these children in fact had been vaccinated in another country introducing the vaccine at a later time point, but it is more likely a recall bias.

Carriage of NmA was still low two years after the introduction of MenAfriVac, and together with the historically low NmA disease incidence post-MenAfriVac vaccination, our data support evidences of prolonged herd immunity established after the mass vaccination campaign [23]. Despite the fact that 70% of the 1-3-year olds were unvaccinated, we did not find any NmA carrier in this age group. In comparison, pre-vaccination NmA carriage prevalence in the 1-3-year-olds was 0.30% [26]. Surprisingly, the single NmA carriage isolate was ST-7 and not ST-2859, the only genotype responsible for NmA disease in Burkina Faso since 2004 [35], and also the only NmA genotype found among carriers in the period 2008–2011 [26],[27]. The child who carried this ST-7 strain had not travelled in the period prior to sampling, but the household had recently received visitors from the Ivory Coast, and colonization of the child by a NmA ST-7 isolate might be a result of transmission from one of the visitors.

The introduction of a monovalent vaccine could result in capsule switch of the ST-2859 clone as a consequence of uptake of genes coding for another capsule without the loss of virulence, as has been described for other Nm clones [40]–[43]. We have previously reported that we did not observe any capsule switch up to 13 months after vaccination [24] and we show here that this observation was still valid two years after vaccination.

The most striking result in this carriage study was the dominance of NmW ST-11 P1.5,2;F1-1, a clone that was responsible for a large NmW outbreak in Burkina Faso in 2000–2002. ST-11 was last detected among patients in Burkina Faso in 2005 and 2006 [35],[44] and was not found among carriers in 2008–2010 [24],[26], before it reappeared during the epidemic season of 2011 [24]. It has previously been suggested that the re-introduction of ST-11 happened from Mali [24], and our results support this hypothesis since the western district of Dandé had significantly higher NmW carriage prevalence when compared to Bogodogo and Kaya. Dandé is close to Mali and NmW caused districts on both sides of the Mali-Burkina Faso border to reach the epidemic threshold in 2012 [36]. A reappearance of NmW ST-11 outbreaks ten years after the 2002 outbreak [9],[45] is consistent with the cyclic waves of meningococcal epidemics [1],[2]. Before MenAfriVac vaccination, the carriage prevalence of NmA was low and the ST-11 clone did not circulate. Thus, it is less likely that a vaccine-induced replacement of NmA ST-2859 with NmW ST-11 could explain the high proportion of NmW carriage and disease two years after vaccination.

The second most frequent clone among carriers and patients was NmX, ST-181 P1.5-1,10-1;F1-31. This same clone caused many cases of meningitis during the epidemic season of 2010, before MenAfriVac introduction [35]. NmX carriage was high in October-November 2010 and decreased throughout 2011 [23]. We show that carriage of NmX continued to decrease in 2012, but that the district of Kaya still had the highest prevalence of NmX. One of the culture-negative invasive NmX isolates had the unusual PorA variant P1.7,16-46. A nested PCR of the MLST loci attributed this isolate to the ST-181 complex, as the other NmX isolates.

Serogroup Y ST-4375 of the ST-23 complex was the dominating carriage clone circulating in 2009, before vaccine introduction [26], but its carriage prevalence gradually declined in 2010–2011 [23] and only 0.6% of the participants carried ST-4375 in 2012. The decline in carriage of the ST-23 complex meningococci is presented in Figure 1. Compared to other virulent clones, the ST-4375 clone is seldom associated with disease and has been only sporadically identified in patients in Burkina Faso in the past ten years, last in 2008 [35]. Although various STs of the ST-23 complex have been causing an increasing number of cases in the US and Europe [46]–[48], only a few NmY cases of disease have been reported in Burkina Faso, or in other countries of the meningitis belt [35].

Since the start of these carriage campaigns, the dominating serogroup has been Y (in 2009), X (in 2010–2011) and W (in 2012) [23],[26]. Carriage prevalence of each of these serogroups varied significantly with age only in the period when that serogroup was dominating. In 2012 when serogroup W dominated, there was a clear peak of carriage prevalence among the 5–9 year old; in 2011 when serogroup X was dominating, maximum carriage was in 5–14 year old [23], and in 2009 when serogroup Y dominated, the peak was in the age group 10–14 years [26].

We have also shown that the same genotypes were found among carriers and patients and that increased carriage of virulent clones was closely associated with increased disease incidence. In Burkina Faso NmX ST-181 carriage prevalence increased during the epidemic season of 2009, especially in the eastern district of Kaya [26] (Figure 1). In the following epidemic season (2010) NmX was found to cause disease and towards the end of 2010, NmX ST-181 carriage was significantly higher in all 3 carriage study sites, and particularly in Kaya [23],[24] (Figure 1). The same pattern was observed for NmW ST-11, first seen among carriers in the western district of Dandé in early 2011, then spreading throughout the country [24]. The following epidemic season NmW ST-11 was the main disease-causing genotype and by the end of 2012, this clone dominated among carriage strains and with the highest prevalence found in Dandé (Figure 1). The introduction of virulent clones in an immunologically naive population seems to be one of the important factors for meningococcal outbreaks [49], together with the inherent properties of the various Nm serogroups and clones, and contact patterns between individuals within the society.

## Conclusions

In this study we found that the carriage prevalence of NmA was still very low in Burkina Faso two years after MenAfriVac mass vaccination, with a single NmA carrier identified among 4969 participants (0.02%). The isolate was assigned to the ST-7, a genotype that has not circulated in Burkina Faso since 2003. The most frequently carried clone was NmW ST-11, a finding consistent with the many cases of NmW meningitis in 2012 and the high proportion of NmW ST-11 among the characterized invasive isolates.

The low NmA carriage prevalence in Burkina Faso two years after MenAfriVac mass vaccination is consistent with the historically low NmA disease incidence and confirms the vaccine’s long term ability to confer herd protection by preventing NmA colonization and transmission. Until MenAfriVac is included in routine immunization programs or is periodically administered through mass vaccination campaigns [50], the unvaccinated population, especially children who were too young to be vaccinated in 2010, will benefit from low NmA transmission in their entourage.

## Authors’ original submitted files for images

Below are the links to the authors’ original submitted files for images. Authors’ original file for figure 1 Authors’ original file for figure 2 Authors’ original file for figure 3
