# Supplementary figures and images for: Persistent low carriage of serogroup A Neisseria meningitidistwo years after mass vaccination with the meningococcal conjugate vaccine, MenAfriVac
Source: BMC Infect Dis. 2014 Dec 4;14:663. doi: 10.1186/s12879-014-0663-4 (PMC4267149; doi:10.1186/s12879-014-0663-4)

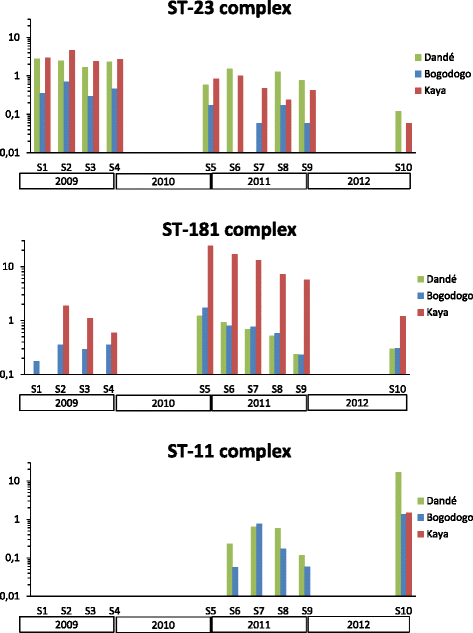

Supplement: Supplementary file 1 — Authors’ original file for figure 1 [file 12879_2014_663_MOESM1_ESM.gif]

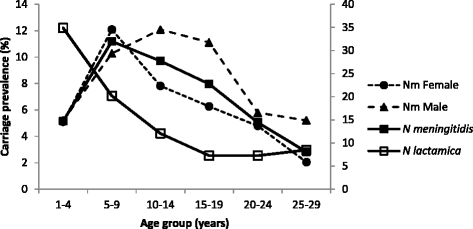

Supplement: Supplementary file 2 — Authors’ original file for figure 2 [file 12879_2014_663_MOESM2_ESM.gif]

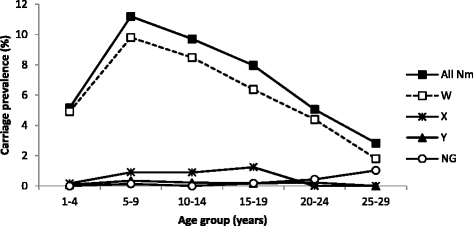

Supplement: Supplementary file 3 — Authors’ original file for figure 3 [file 12879_2014_663_MOESM3_ESM.gif]
